# Comparison of five different Light-Cured Pulp Capping Materials to a Hydraulic Calcium Silicate Cement: A Long-Term Physicochemical Study

**DOI:** 10.1016/j.identj.2026.109554

**Published:** 2026-05-05

**Authors:** Laurentia Schuster, Till Dammaschke, Roxana Trusca, Ludmila Motelica, Benjamin Ehmke, Denisa Ficai, Anton Ficai

**Affiliations:** a Department of Periodontology and Operative Dentistry, University of Münster, Münster, Germany; b Faculty of Chemical Engineering and Biotechnologies, Department of Science and Engineering of Oxide Materials and Nanomaterials, National University of Science and Technology POLITEHNICA Bucharest, Bucharest, Romania; c National Research Center for Micro and Nanomaterials, Faculty of Chemical Engineering and Biotechnologies, National University of Science and Technology POLITEHNICA Bucharest, Bucharest, Romania; d National Centre for Food Safety, National University of Science and Technology POLITEHNICA Bucharest, Bucharest, Romania; e Department of Inorganic Chemistry, Physical Chemistry and Electrochemistry, Faculty of Chemical Engineering and Biotechnologies, National University of Science and Technology POLITEHNICA Bucharest, Bucharest, Romania; f Academy of Romanian Scientists, Bucharest, Romania

**Keywords:** Vital pulp therapy, Pulp capping, Biodentine, Light-cured pulp capping materials, Material properties

## Abstract

**Objectives:**

Hydraulic calcium silicate cements (HCSCs) are the gold standard for vital pulp therapy, although they have a prolonged setting time. Light-cured flowable composite resins containing calcium silicate or calcium hydroxide powder are promoted as an alternative. The aim of this study was to compare the physicochemical properties of 5 light-cured pulp capping materials (LCPCMs) (Calcimol LC, TheraCal LC, ReViCal, MTA PulpCap, and Pulprotec MTA) with HCSC (Biodentine).

**Methods:**

Cylindrical specimens (51.472 mm^3^; n = 13/material/time) were stored in 5 mL Hanks’ Balanced Salt Solution in a Thermoshaker at 37 °C with constant shaking. After 1, 3, 7, 14, 28, and 180 days of immersion, the pH value and conductivity of the solution, the solubility of the samples, and the surface properties of the specimens (scanning electron microscopy, energy dispersive x-ray, x-ray diffraction, Fourier transformation infrared spectroscopy) were analysed. Statistical analysis was carried out using the Fisher exact test and the 2-sample *t* test in addition to 1-way analysis of variance.

**Results:**

The pH value of Biodentine was significantly higher than that of the LCPCM at every time point examined (*P* < .05). On scanning electron microscopy, hydroxyapatite-like structures were visible on the surface of Biodentine after 7 days, whereas this was not the case for the LCPCM. The energy dispersive x-ray revealed that significantly more calcium was found on the surface of Biodentine than in the LCPCM (*P* < .05). X-ray diffraction was able to detect hydroxyapatite on the surface of Biodentine, while all materials showed calcite on their surfaces. Fourier transformation infrared spectroscopy confirmed these findings and showed polymeric structures in the LCPCM.

**Significance:**

The physicochemical properties of HCSC (Biodentine) examined in this study are superior to those of LCPCM.

## Introduction

Vital pulp therapy (VPT) is the treatment of choice for an exposed, vital pulp. With the correct indication, VPT has high clinical success rates.^1^, ^2^, ^3^, ^4^ Most important for successful VPT is pulp tissue free of infection with microorganisms and the correct choice of pulp capping material. The material applied to the exposed pulp seals it from the oral cavity, preventing contact of microorganisms or restoration materials with the pulp. Pulp capping materials are required to have high biocompatibility, cause no toxic effects on pulpal cells, and have the ability to induce the formation of reparative hard tissue.^5^

Hydraulic calcium silicate cements (HCSCs) are the gold standard in VPT. They have an alkaline pH value and release calcium and silicon ions.^6^ Calcium ions are an important modulator of cell differentiation.^7^, ^8^, ^9^ Together with hydroxyl ions, they enhance the production of BMP-2 and osteopontin in pulpal cells, as well as the activation of alkaline phosphatase and the release of growth factors from dentine.^10^^,^^11^ All of these are indispensable for the mineralisation process. In addition, an alkaline pH value has a disinfecting effect on surrounding tissue.^12^

All available HCSCs share the disadvantage of a relatively long setting time of about 15 minutes^6^ to several hours.^13^ Hence, multiple light-cured flowable composite resins containing calcium hydroxide or calcium silicate powder have been introduced into the dental market. It is advertised that these light-cured pulp capping materials (LCPCMs) combine the advantages of a composite resin filling material with those of HCSCs.

Investigations of the physicochemical properties of the resin-based LCPCMs were conducted on various aspects, but with many new materials being introduced, not all of them have been investigated yet. When stored in distilled water^14^, ^15^, ^16^ or simulated body fluids,^17^^,^^18^ LCPCMs induce a lesser pH increase of the storage solution than HCSCs and release fewer calcium ions into the storage solution.^15^^,^^17^, ^18^, ^19^, ^20^, ^21^, ^22^, ^23^ Surface analyses by scanning electron microscopy (SEM), energy dispersive x-ray (EDS), x-ray diffraction (XRD) comparing HCSCs and LCPCMs revealed that on the surface of HCSCs, calcium hydroxide and tricalcium silicate could be found, whereas calcium hydroxide could not be detected on the surface of LCPCMs.^14^^,^^21^^,^^22^

LCPCMs are promoted by the manufacturers to be a suitable alternative to HCSCs in VPT, and new materials of this material class are regularly introduced. Still, whether LCPCMs have a negative impact on the surrounding tissues and, respectively, can ensure proper pulp healing needs further investigation. This includes the assessment of their physicochemical properties to establish whether the LCPCMs have the same abilities as hydraulic calcium silicate cements.

The aim of the present study was to assess the physicochemical properties and the surface properties of 5 different LCPCMs in comparison to the HCSC Biodentine. The LCPCMs used in this study include 3 recently introduced materials (ReViCal, MTA PulpCap, and Pulprotec MTA). Therefore, this is the first time that the physicochemical properties and the surface properties of these materials have been assessed.

## Material and methods

### Pulp capping materials and study design

In this study, the physicochemical properties of 5 LCPCMs and 1 HCSC were investigated (Table 1): Biodentine (BD; Septodont) as a positive control, Calcimol LC (CLC; VOCO), TheraCal LC (TLC; BISCO), ReViCal (RVC; R-Dental), and MTA PulpCap and Pulprotec MTA (MPC and PPM; both Cumdente).

**Table 1:** Pulp capping materials investigated, ingredients according to the manufacturers’ SDS.

Product name	Material	Ingredients	Manufacturer	Indications
Biodentine	Hydraulic calcium silicate cement Powder and liquid	Powder: tricalcium silicate, zirconium oxide, calcium oxide, calcium carbonate Liquid: aqueous calcium chloride and polycarboxylate	Septodont	Direct and indirect pulp Capping, pulpotomy
Calcimol LC	Flowable composite resin 1-component-system Light-curing	Calcium hydroxide, fumed silica, UDMA, TEGDMA	VOCO	Indirect pulp capping
TheraCal LC	Flowable composite resin 1-component-system Light-curing	Portland cement type III, fumed silica, Bis-GMA, polyglycol dimethacrylate, barium zirconate	BISCO	Direct and indirect pulp capping
ReViCal	Flowable composite resin 1-component-system Light-curing	Mixture of various mineral oxides and methacrylates: mineral oxides GHS05, GHS07, H315, H317, H318; methacrylates GHS07, H315, H319, H335, H317	R-dental	Direct and indirect pulp capping
MTA PulpCap	Flowable composite resin 1-component-system Light-curing	Mixture of various mineral oxides and methacrylates: mineral oxides GHS05, GHS07, H315, H318, H317; methacrylates GHS07, H315, H319, H335, H317	Cumdente	Direct and indirect pulp capping
Pulprotec MTA	Flowable composite resin 1-component-system Light-curing	Mixture of various mineral oxides and methacrylates: mineral oxides GHS05, GHS07, H315, H318, H317; methacrylates GHS07, H315, H319, H335, H317	Cumdente	Indirect pulp capping

SDS, Safety Data Sheet.

Biodentine was mixed according to the manufacturer’s recommendations without prior treatment; the other materials (ReViCal, TheraCal LC, MTA PulpCap, Pulprotec MTA, and Calcimol LC) were light-cured without prior treatment. The LCPCMs were pressed into moulds (6.4 mm diameter; 1.6 mm height; 51.472 mm^3^ volume), covered from both sides with glass plates, and light-cured according to the manufacturer’s protocols for 20 seconds (CLC, TLC) or, respectively, 40 seconds (RVC, MPC, PPM) on both sides, each with a light-curing device at 1000 mW/cm^2^ and a 460- to 490-nm wavelength (SmartLite Focus; Dentsply Sirona) at room temperature. BD was mixed according to the manufacturer’s recommendations, pressed into moulds, covered from both sides with glass plates, and left to set for 15 minutes at room temperature.

After curing, the obtained test bodies were stored in 5 mL Hanks’ Balanced Salt Solution (HBSS, H6648; Sigma-Aldrich) in sealed test tubes at 37 °C and a constant shaking rate (25 rpm) in a Thermoshake (Laboshake; C. Gerhardt Analytical Systems). The pH value and the conductivity of the solution, the mass change and water absorption of the samples, and the surface modification of the samples (SEM, EDS, XRD, and Fourier transformation infrared spectroscopy [FTIR]) were examined at different time points. In total, 78 experimental samples per material were produced (n = 13 per time point investigated).

### Material assessments in simulated body fluid

Prior to being immersed in liquid, the samples were weighed 3 times each using an analytical scale (AS 220.R2; RADWAG) to obtain their initial weight. The test bodies (13 per time point investigated) were then stored in 5 mL HBSS in test tubes sealed with screw lids and parafilm at 37 °C and a constant shaking rate of 25 rpm in a Thermoshaker. During storage time, the HBSS was not refreshed. At baseline (t = 0) and after 6 different time points (1, 3, 7, 14, 28, and 180 days), the pH value and the conductivity of the solution were measured using a pH and conductivity meter (inoLab Multi 9630 IDS; Xylem Analytics). The measurements were carried out at room temperature immediately after removing the test tubes from the Thermoshaker and repeated 3 times each. The test bodies were removed from the test tubes, carefully rinsed with distilled water, and blotted dry with filter paper. They were then weighed 3 times each again to obtain their wet weight. The samples were then placed in a heating cabinet (Universal oven UF 55plus; Memmert GmbH) at 40 °C and 0% humidity for 24 hours to dry completely. The samples were again weighed 3 times each to obtain their final dry weight. The mass change of the samples was calculated using the following formula:

$$mass\;change\;\left[\%\right]=\frac{dry\;weight\;at\;time\;t-initial\;weight}{initial\;weight}*100$$

The water absorption of the samples was calculated using the following formula:

$$water\;absorption\;\left[\%\right]=\;\frac{wet\;weight\;at\;time\;t-dry\;weight\;at\;time\;t}{dry\;weight\;at\;time\;t}*100$$

### SEM, EDS, and XRD analysis

After the test specimens were removed from the HBSS and dried as described above, their surfaces were analysed by SEM, EDS, and XRD at baseline (t = 0) and at 4 different time points (t = 7 days, t = 14 days, t = 28 days, and t = 6 months; n = 3 per time point investigated). All the analyses were carried out at room temperature. For the SEM and EDS analysis, the samples were coated with gold for 60 seconds prior to imaging. The SEM and EDS investigation of the samples was carried out using the QUANTA INSPECT F50 scanning electron microscope (FEI Company) equipped with a field emission electron gun (FEG) with a resolution of 1.2 nm and an EDS with MnK resolution of 133 eV at a voltage of 30 kV, a tilt of 0, and a take-off of 34.3 Amp.

The XRD analysis was carried out using the SHIMADZU XRD 6000 diffractometer (Shimadzu). For all analyses, the Cu K_α_ radiation from a Cu x-ray tube was used at a voltage of 40 kV and a current of 30 mA. The divergence and scatter slits were set at an angle of 1.00000 degrees, and the receiving slit was set at 0.30000 mm. The samples were scanned at a drive axis of Theta-2-Theta and a scan range of 10.000 to 70.000 in a continuous scan mode. The scan speed was set to 2.0000 degrees per minute, the sampling pitch to 0.0200 degrees, and the preset time to 0.6 seconds.

### FTIR and FTIR-map analysis

After the test specimens were removed from the HBSS and dried as described above, their surfaces were analysed by infrared spectroscopy and FTIR and FTIR microscopy at reference (t = 0) and after the abovementioned time points of immersion in HBSS (n = 3 per time point investigated). A Nicolet iN10 MX infrared imaging microscope (Thermo Fisher Scientific) was used to record the spatial distribution maps of the components. All measurements were performed at room temperature using the attenuated total reflection (ATR) accessory with the detector cooled with liquid nitrogen. Each spectrum was recorded between 675 and 4000 cm^–1^ with a resolution of 4 cm^–1^.

A Nicolet iS50 R infrared imaging microscope (Thermo Fisher Scientific) equipped with an ATR accessory was used to record FTIR spectra. Spectra were recorded at room temperature, with each spectrum representing the average of 32 scans, from 400 to 4000 cm^–1^, with a resolution of 4 cm^–1^.

### Statistical analysis

The acquired data were prepared in Excel 2020 (Microsoft) and formatted to be read by Excel’s “XL Toolbox NG” and RStudio (RStudio, Posit PBC [formerly RStudio Inc.]). Statistical analysis for each investigation was carried out using 1-way analysis of variance (ANOVA) with the modified Levene test, with *P* < .05. The post hoc Bonferroni-Holm test was used to evaluate pH, conductivity, and solubility. The EDS investigation was additionally evaluated by the χ^2^ test and the post hoc Fisher exact test. To double-check the results, a 2-sample *t* test was used.

## Results

### Material evaluation in simulated body fluids

**Fig. 1:**
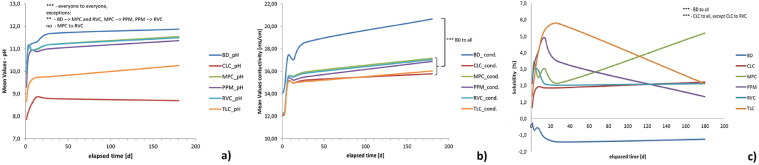
(a) Mean pH values after 1 to 180 days. Statistically significant differences (*P* < .05) between the pH values of the materials are marked by asterisks: *** (highly significant) and ** (significant). (b) Mean values of conductivity after 1 to 180 days, with significant differences between BD and the LCPCMs marked by asterisks: *** (highly significant). (c) Mean mass change of the materials from 1 to 180 days. The negative bars for BD mean that the material has a solubility of about 1.3% (highest measured value at t_5_ = 28 days), while the positive bars of the LCPCMs mean that they absorbed water during the investigation period, resulting in weight gain; thus, solubility of the materials could not be found. Statistically significant differences are marked by asterisks: ***(highly significant). For all materials, n = 13 samples per time point (1, 3, 7, 14, 28, 180 days) were examined. BD, Biodentine; LCPCM, light-cured pulp capping materials.

The pH values of the solution determined for BD were pH 10.36 ± 0.75 after 24 hours and pH 11.87 ± 0.13 after 6 months. The lowest pH values among the LCPCMs were determined for CLC, which were pH 7.86 ± 0.40 after 24 hours and pH 8.69 ± 0.53 after 6 months. The highest pH values among the LCPCMs were found for MPC and RVC with pH 9.90 ± 0.21 and pH 9.99 ± 0.29, respectively, after 24 hours and pH 11.54 ± 0.09 and pH 11.49 ± 0.07, respectively, after 6 months. The pH values of MPC and TLC were nearly identical, as the green and turquoise curves in Figure 1a depict. The statistical analysis showed that the differences between BD and all LCPCMs, as well as the differences between the LCPCMs, were statistically significant at each examination time (*P* < .05; Figure 1a).

The ionic conductivity of aqueous samples characterises the ionic content of aqueous solutions as a sum parameter, it being known that the contribution of each ion is proportional to its molar ionic conductivity. It is worth mentioning that exceptionally high molar ionic conductivity is specific for H^+^ (∼350 S**^.^**cm/mol) and HO^–^ (∼200 S**^.^**cm/mol), while for the other ions, it is usually below 100 S**^.^**cm/mol. The measurement of the conductivity of the HBSS solution showed at each examination time, the solution in which BD samples were stored had a higher conductivity, reaching 20.66 ±1.79 mS/cm after 6 months, than those in which LCPCMs were stored, reaching values only between 15.79 ± 0.35 mS/cm and 17.17 ± 0.35 mS/cm. Figure 1b shows that the conductivity curves over time of the LCPCMs are very similar, with those of MPC, PPM, and RVC being nearly identical and the curves of CLC and TLC also being nearly identical. This means that the conductivity of the storage liquid behaves similarly, even if the pH evolution of the materials is quite different. Considering all these data, the ionic release has to be different. The statistical analysis showed that the difference between BD and all LCPCMs was statistically significant at each examination time (*P* < .05), while the differences between the LCPCMs were not statistically significant.

The mass change of the materials determined shows a difference between BD and LCPCMs. For BD, a mass loss of approximately 1.26% after 180 days was found, leading to a decrease in mass (see blue curve in Figure 1c). For LCPCMs, the determined mass change was overall positive. The curves in Figure 1c show that all 5 investigated LCPCMs gained mass, although the curves differed over time. CLC exhibited a mass gain of 2.22% after 180 days, MPC had a mass gain of 5.18% after 180 days, PPM had a mass gain of 1.33% after 180 days, and RVC and TLC had a mass gain of 2.12% after 180 days. When examining water absorption, BD consistently absorbed between 2.45% and 4.46% of its weight in water at all time points examined. In contrast, the LCPCMs had greater water absorption over time, increasing from 1.86% to 9.86% at 1 day to 5.15% to 18.19% at 180 days. Thus, the solubility of BD can be derived from the change in weight, whereas this is not the case for all LCPCMs. The difference between BD and all LCPCM was statistically significant (*P* < .05) in all cases. Macroscopically, a white precipitate could be seen in the sample tubes after 28 days and 6 months.

### SEM and EDS analysis

**Fig. 2:**
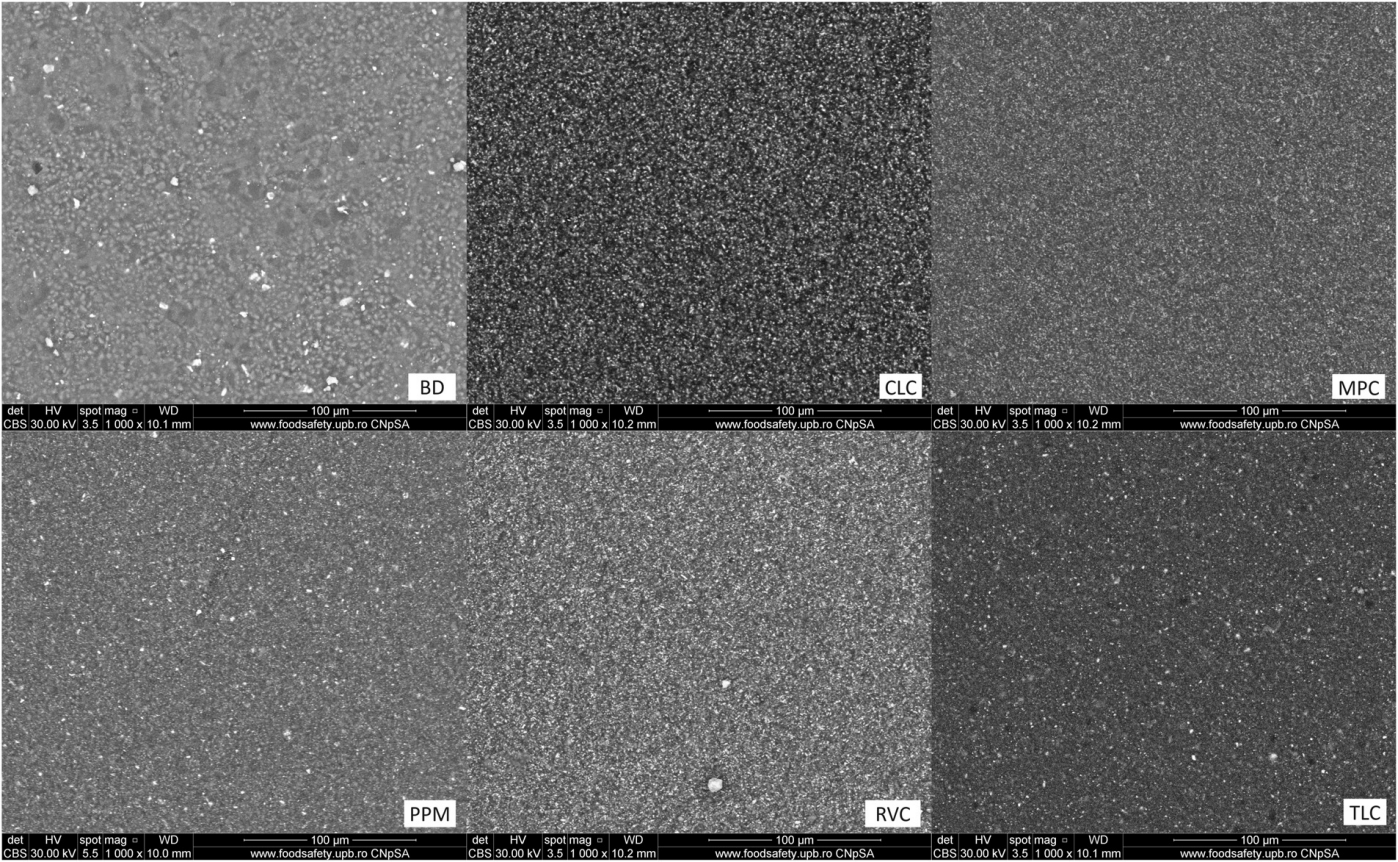
Representative scanning electron microscopy images of each material (n = 3) at baseline (1000×).

**Fig. 3:**
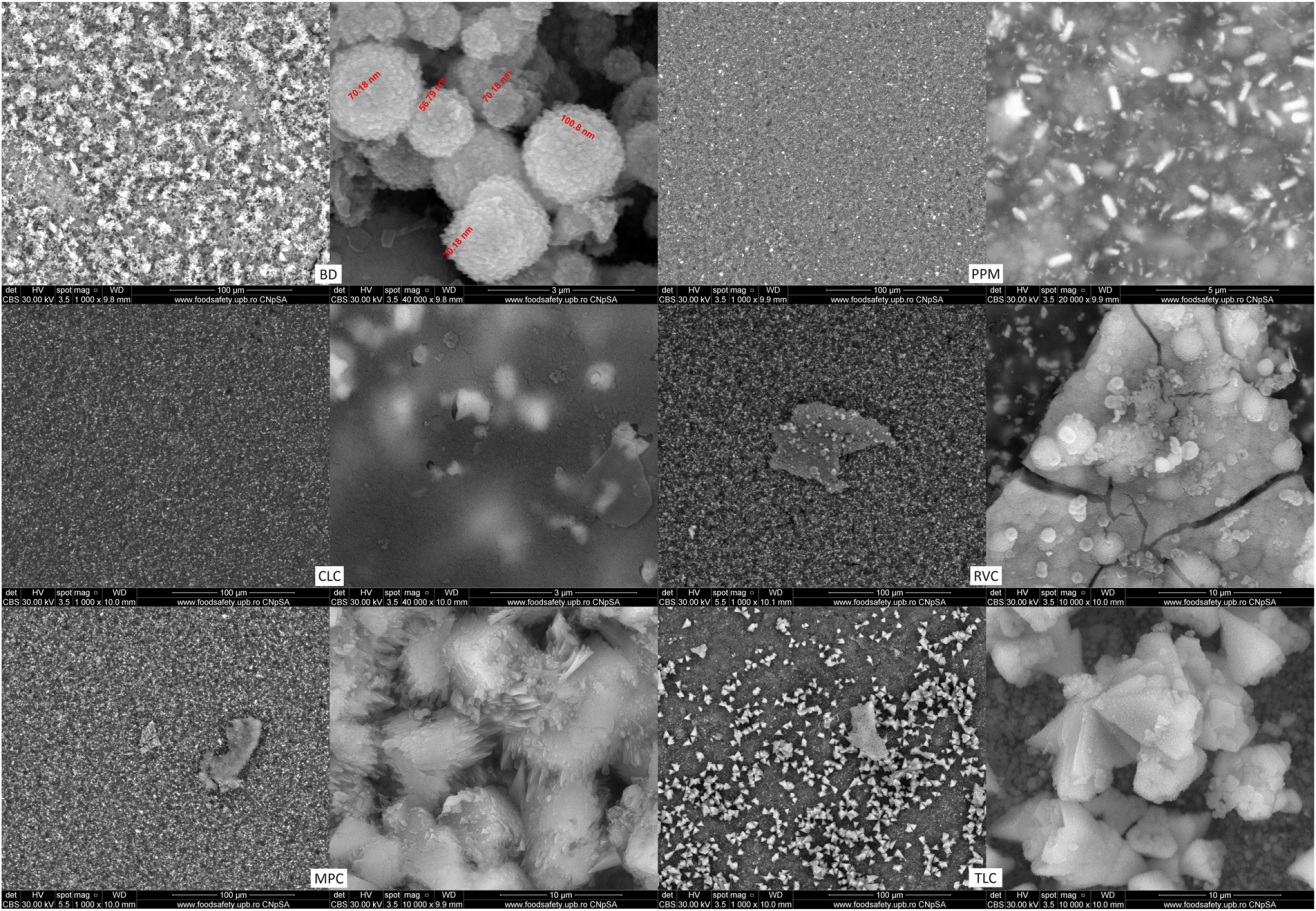
Representative SEM images of samples (n = 3) at t = 7 days. BD after 7 days (1000× and 40,000×; red numbers mark nanostructures from 56.79 to 100.8 nm), CLC after 7 days (1000× and 40,000×), MPC after 7 days (1000× and 10,000×), PPM after 7 days (1000× and 20,000×), RVC after 7 days (1000× and 10,000×), and TLC after 7 days (1000× and 10,000×). For MPC and RVC, different magnifications were chosen to show that the deposits seem to build a second layer on top of the material’s surface and do not cover the whole surface. BD, Biodentine; CLC, Calcimol LC; MPC, MTA PulpCap; PPM, Pulprotec MTA; RVC, ReViCal; SEM, scanning electron microscopy; TLC, TheraCal LC.

**Fig. 4:**
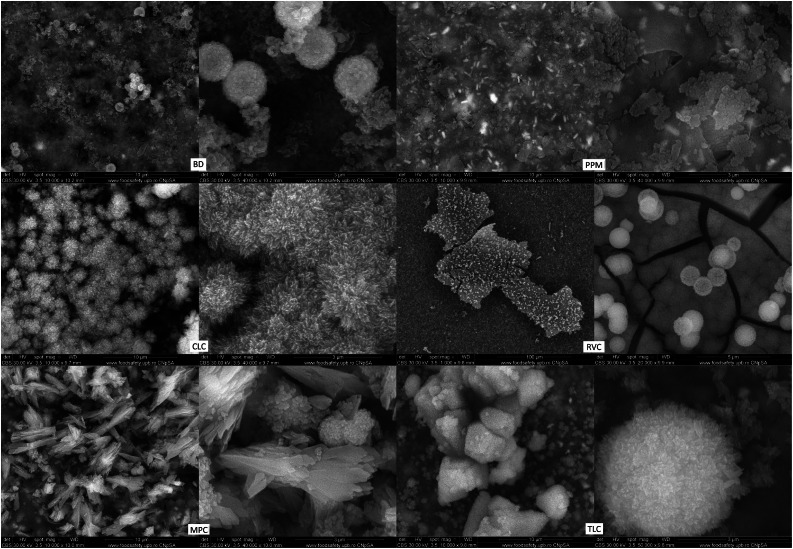
Representative SEM images of samples (n = 3) at t = 14 days. BD after 14 days (10,000× and 40,000×), CLC after 14 days (10,000× and 40,000×), MPC after 14 days (10,000× and 40,000×), PPM after 14 days (10,000× and 40,000×), RVC after 14 days (1000× and 20,000×), and TLC after 14 days (10,000× and 50,000×). For RVC, different magnifications were chosen to show that the deposits seem to build a second layer on top of the material’s surface and do not cover the whole surface. BD, Biodentine; CLC, Calcimol LC; MPC, MTA PulpCap; PPM, Pulprotec MTA; RVC, ReViCal; SEM, scanning electron microscopy; TLC, TheraCal LC.

**Fig. 5:**
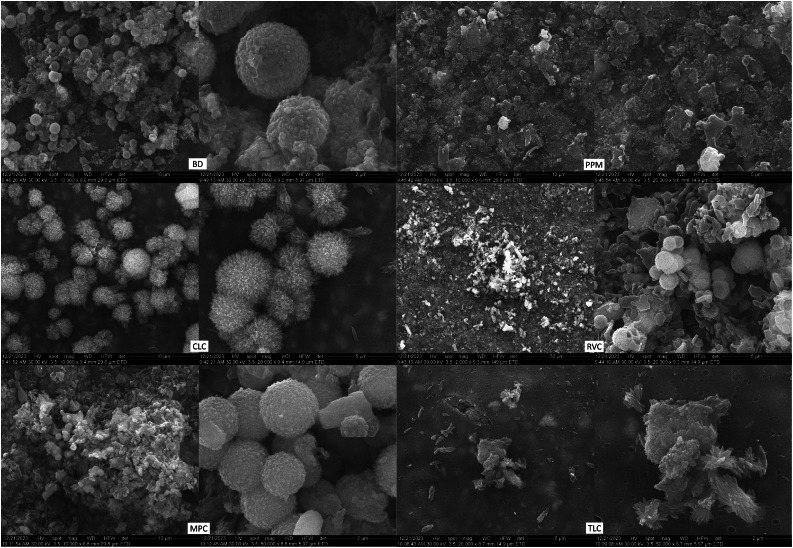
Representative SEM images of samples (n = 3) at t = 28 days. BD after 28 days (10,000× and 50,000×), CLC after 28 days (10,000× and 20,000×), MPC after 28 days (10,000× and 50,000×), PPM after 28 days (10,000× and 20,000×), RVC after 28 days (1000× and 20,000×), and TLC after 28 days (20,000× and 50,000×). BD, Biodentine; CLC, Calcimol LC; MPC, MTA PulpCap; PPM, Pulprotec MTA; RVC, ReViCal; SEM, scanning electron microscopy; TLC, TheraCal LC.

**Fig. 6:**
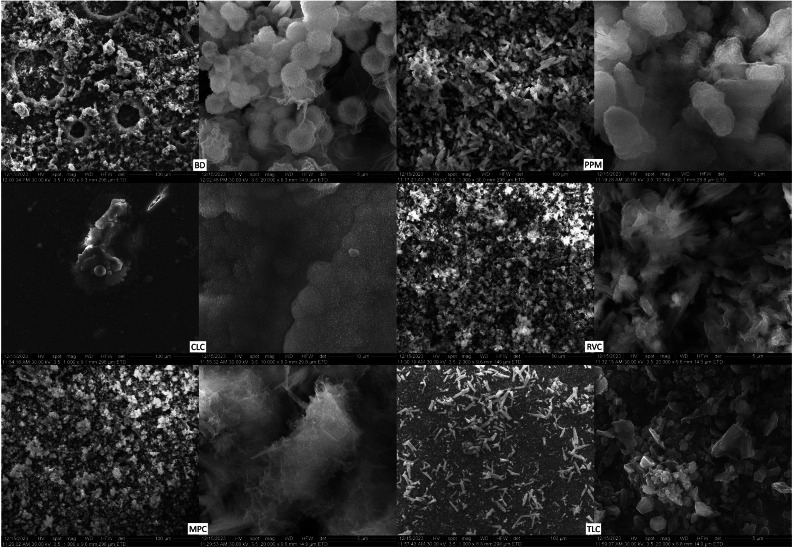
Representative SEM images of samples (n = 3) at t = 180 days. BD after 180 days (1000× and 20,000×), CLC after 180 days (1000× and 10,000×), MPC after 180 days (1000× and 20,000×), PPM after 180 days (1000× and 10,000×), RVC after 180 days (2000× and 20,000×), and TLC after 180 days (1000× and 20,000×). BD, Biodentine; CLC, Calcimol LC; MPC, MTA PulpCap; PPM, Pulprotec MTA; RVC, ReViCal; SEM, scanning electron microscopy; TLC, TheraCal LC.

The SEM analysis revealed that the sample surface of the BD reference sample prior to immersion in the simulated body fluid (Figure 2) corresponded to the typical cement surface. It had a smooth, even surface with cavities interspersed with many small cement particles and radio-opacity molecules (zirconium). The surface of the samples of the LCPCM reference, prior to immersion in simulated body fluid (Figure 2), on the other hand, showed the surface typical of an organic polymer composite resin structure. The LCPCM also had a smooth surface, but this surface was formed by the organic matrix of the composite resins. The organic matrix was interspersed with cement particles and radio-opacity molecules (zirconium, barium, or ytterbium). After just 7 days of immersion in HBSS, typical hydroxyapatite-like precipitates consisting of many blossom-like structures were visible on the surface of the BD when analysed by scanning electron microscopy (Figure 3). The precipitates seemed to grow out of and directly on the surface of the BD as a consequence of Ca^2+^ dissolution and precipitation in the presence of phosphate, at a proper pH (pH of the solution reached 11 within 1 day, as visible from Figure 1a). The organic matrix of the LCPCMs, on the other hand, appeared to be washed out and blurred. The surfaces of CLC and PPM were still bare and uncovered (Figure 3). On the surface of MPC and RVC, isolated scattered deposits were visible, which appeared as pointed and jagged structures at higher magnification (Figure 3)—RVC was also more active than the other LCPCMs, as revealed by the HBSS evaluation (pH, conductivity, and mass change, as depicted in Figure 1). The deposits seemed to build up a second layer on top of the sample surface. Many scattered deposits with crystalline shapes were visible on the surface of the TLC, which seemed to be salt-like structures on closer inspection (Figure 3). The deposits on the surface of MPC, RVC, and TLC were structured like salt crystals and corresponded most closely to the compounds halite (NaCl), calcium oxide (CaO), and calcite (CaCO_3_) detected in the XRD. After 14 and 28 days, as well as after 6 months, the aforementioned structures became denser on the surface of the samples. Crystalline deposits could now also be seen on the surfaces of CLC and PPM. An organisation of the hydroxyapatite-like deposits on the surface of the BD, as well as the salt-like crystal structures on the surface of the LCPCMs in several layers, became visible from about 14 days onwards (see Figure 4). Occasionally, individual blossom-like spheres were visible between the salt-like crystals on the surface of MPC and RVC after 28 days (see Figure 5) but not after 6 months (see Figure 6).

**Fig. 7:**
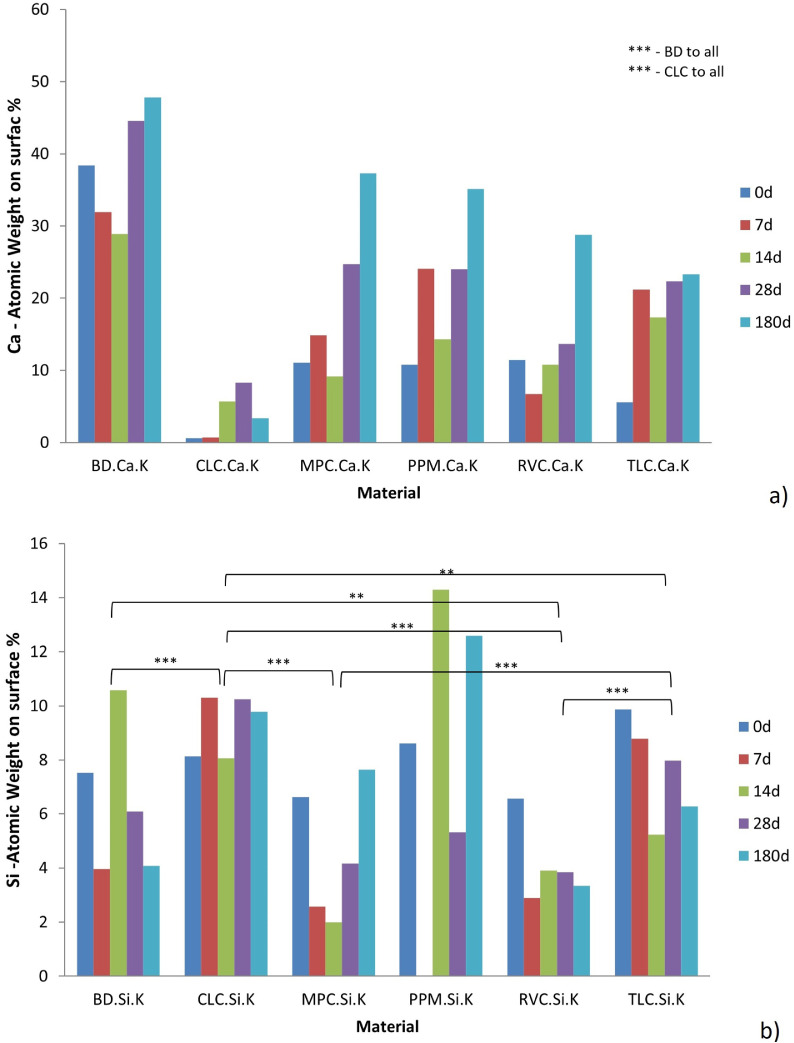
(a) Bar chart of the atomic weight (%) of calcium on the surface of the material samples at different time points investigated. Statistically significant differences are marked by asterisks: *** (highly significant). (b) Bar chart of the atomic weight (%) of silicon on the surface of the material samples at different time points investigated. For all materials, n = 3 samples per time point (7, 14, 28, 180 days) were examined. Statistically significant differences are marked by asterisks: *** (highly significant) and ** (significant).

The EDS analysis of the sample surface showed that significantly more calcium was found on the surface of the BD samples at all time points examined (see Figure 7a). The difference between BD and all LCPCMs was statistically significant (*P* < .05). The amounts of silicon detectable on the sample surface were similar for all materials, but the values varied between the individual time points (see Figure 7b). PPM had the highest measured silicon values at 14 days and 180 days—probably because of the preferential dissolution of other elements from the sample. The total number of elements detected on the sample surface in the EDS is shown in Table 2, and the figures in brackets refer to the atomic weight percentage (at%) of the detected elements. Monitoring only phosphate, which was deposited from the HBSS during the immersion, thus proving the formation of the phosphate phase (calcium phosphates—CaPs, in general), it is obvious that BD developed the highest amount of CaPs, the content of P increased from 0 to 2.02 at%, and in all other cases, the P content was below 0.93 at%. For instance, for TLC, the evolution of the P content was from 0.17 at% for the reference sample and reached 0.93 at% after 6 months of immersion in HBSS—even if, at 14 days, the P content was 2.11 at%. Moreover, for RVC, the final content of P was 0.22 at%, which was even lower than the content from the reference sample. Thus, the complex evolution of the elemental composition was highlighted also by EDS (Figure 7 and Table 2), additionally supporting the complexity of the processes that occurred on the surface of the BD and LCPCMs.

**Table 2:** Atomic weight percentage of all elements detected in the EDS analysis on the sample surface at baseline and after 7 days, 14 days, 28 days, and 6 months (180 days).

	BD	CLC	MPC	PPM	RVC	TLC
Baseline	O (72.35), Si (5.88), Ca (20.98), Cl (0.79)	O (31.33), Si (4.48), Ca (0.22), C (54.6), Al (0.79), S (0.86), Ba (0.7), Na (0.99), K (0.07), N (5.95)	O (43.45), Si (3.99), Ca (4.67), C (45.42), Al (0.64), S (1.1), P (0.72)	O (46.12), Si (5.07), Ca (4.45), C (43.97), Yb (0.39)	O (43.34), Si (4.0), Ca (4.87), C (45.15), Al (0.58), S (1.26), P (0.81)	O (41.2), Si (5.58), Ca (2.21), C (48.91), Al (1.26), Ba (0.7), P (0.17), Mg (0.31), Sr (0.21), Zr (0.14)
7 days	O (77.56), Si (2.93), Ca (16.55), P (2.96)	O (32.4), Si (5.95), Ca (0.29), C (56.11), Al (1.31), S (1.39), Ba (0.97), Na (1.42), K (0.16)	O (58.09), Si (1.7), Ca (6.86), C (27.15), S (1.13), Ba (0.77), P (0.43)	O (70.47), Ca (10.71), C (18.82)	O (35.94), Si (1.65), Ca (2.75), C (56.6), Al (0.64), S (1.46), Ba (0.99)	O (65.33), Si (6.4), Ca (10.83), C (10.81), Al (0.9), P (5.07), Sr (0.5), Au (0.16)
14 days	O (64.71), Si (7.59), Ca (14.54), C (11.76), P (1.4)	O (44.39), Si (4.87), Ca (2.4), C (43.38), Al (0.96), S (0.99), Ba (0.77), P (2.23)	O (49.33), Si (1.18), Ca (3.8), C (43.09), S (1.26), Ba (0.85), P (0.49)	O (24.37), Si (13.88), Ca (9.73), C (32.47), Al (14.45), Ba (2.84), Yb (2.25)	O (48.78), Si (2.3), Ca (4.46), C (43.16), Ba (068), P (0.63)	O (58.56), Si (3.37), Ca (7.84), C (26.43), Al (1.08), P (2.11), Sr (0.6)
28 days	O (38.13), Si (4.74), Ca (24.36), C (27.92), P (1.67), Al (0.36), Na (1.97), Cl (0,85)	O (18.6), Si (6.76), Ca (3.82), C (63.02), Al (0.77), S (1.32), Ba (2.04), Na (0.48), P (3.12), Cl (0.07)	O (27.87), Si (2.95), Ca (12.29), C (52.92), Al (0.71), Ba (2.02), P (1.23)	O (24.87), Si (3.63), Ca (11.48), C (56.8), Al (1.47), Ba (0.39), Na (0.32), Cl (0.04)	O (19.43), Si (2.4), Ca (5.97), C (66.94), Al (0.51), S (1.74), Ba (1.59), P (1.44)	O (41.2), Si (5.58), Ca (2.21), C (48.91), Al (1.26), Ba (0.31), P (0.67)
6 months	O (44.08), Si (3.19), Ca (26.29), C (24.41), P (2.02)	O (15.7), Si (5.75), Ca (1.39), C (72.81), Al (0.82), S (1.22), Ba (1.66), P (0.63)	O (42.51), Si (6.02), Ca (20.57), C (27.24), Al (0.85), S (0.97), Ba (0.93), P (0.92)	O (45.69), Si (10.01), Ca (19.58), C (20.16), Al (1.87), Ba (0.27), P (0.57), Na (1.19), Yb (0.28), Cl (0.38)	O (32.92), Si (2.26), Ca (13.64), C (48.64), Al (0.45, S (0.93), Ba (0.94), P (0.22)	O (26.9), Si (3.96), Ca (10.31), C (56.1), Al (0.69), P (0.93), Sr (1.11)

BD, Biodentine; CLC, Calcimol LC; EDS, energy dispersive x-ray; MPC, MTA PulpCap; PPM, Pulprotec MTA; RVC, ReViCal; SEM, scanning electron microscopy; TLC, TheraCal LC.

### XRD, FTIR, and FTIR-map analysis

**Fig. 8:**
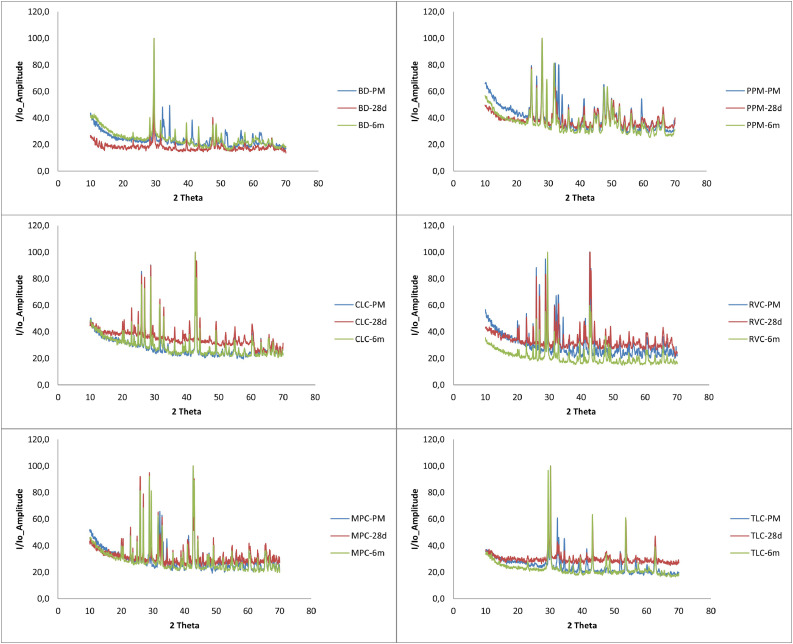
The x-ray diffraction spectra of the material samples at baseline, 28 days, and 6 months (180 days). For all materials, n = 3 samples were examined per time point.

The immersion of the samples in HBSS was also evaluated by XRD, and changes were observed on the surface. The results obtained in the XRD analysis of the sample surfaces agreed with the results of the EDS analysis. Figure 8 shows the XRD diffraction patterns for all evaluated materials, including the reference material before immersion, as well as the materials immersed in HBSS for 28 days and 6 months, respectively.

BD, according to the XRD pattern, was mainly composed of calcium carbonate (calcite) and a mixture of calcium silicates (mainly C_2_S), which predominantly transformed into calcium phosphates as a consequence of immersion in HBSS. For CLC, immersion in HBSS led to slight surface carbonation (calcite peaks 00-083-1762 appeared) and a limited amount of apatite. For BD, the XRD spectra of the sample evaluated after 6 months of immersion in HBSS were only marginally changed compared to the reference XRD diffractogram. The MPC and PPM samples exhibited a similar behaviour to BD; the calcium silicate peaks were practically disappearing while the calcite peaks increased in intensity. For RVC, the carbonation process was more important, with the calcite peaks being increased, thus becoming the most important peak, while for TLC, this peak had almost the same intensity as in RVC.

Based on all these analyses, it can be concluded that the crystalline silicate phases of all the samples were resorbed, and an apatite phase was obtained for BD, CLC, MPC, PPM, and RVC. For TLC, the formation of the apatite phase was difficult to identify.

In the FTIR-map analysis, strong changes were observed for all the analysed samples in all cases.

**Fig. 9:**
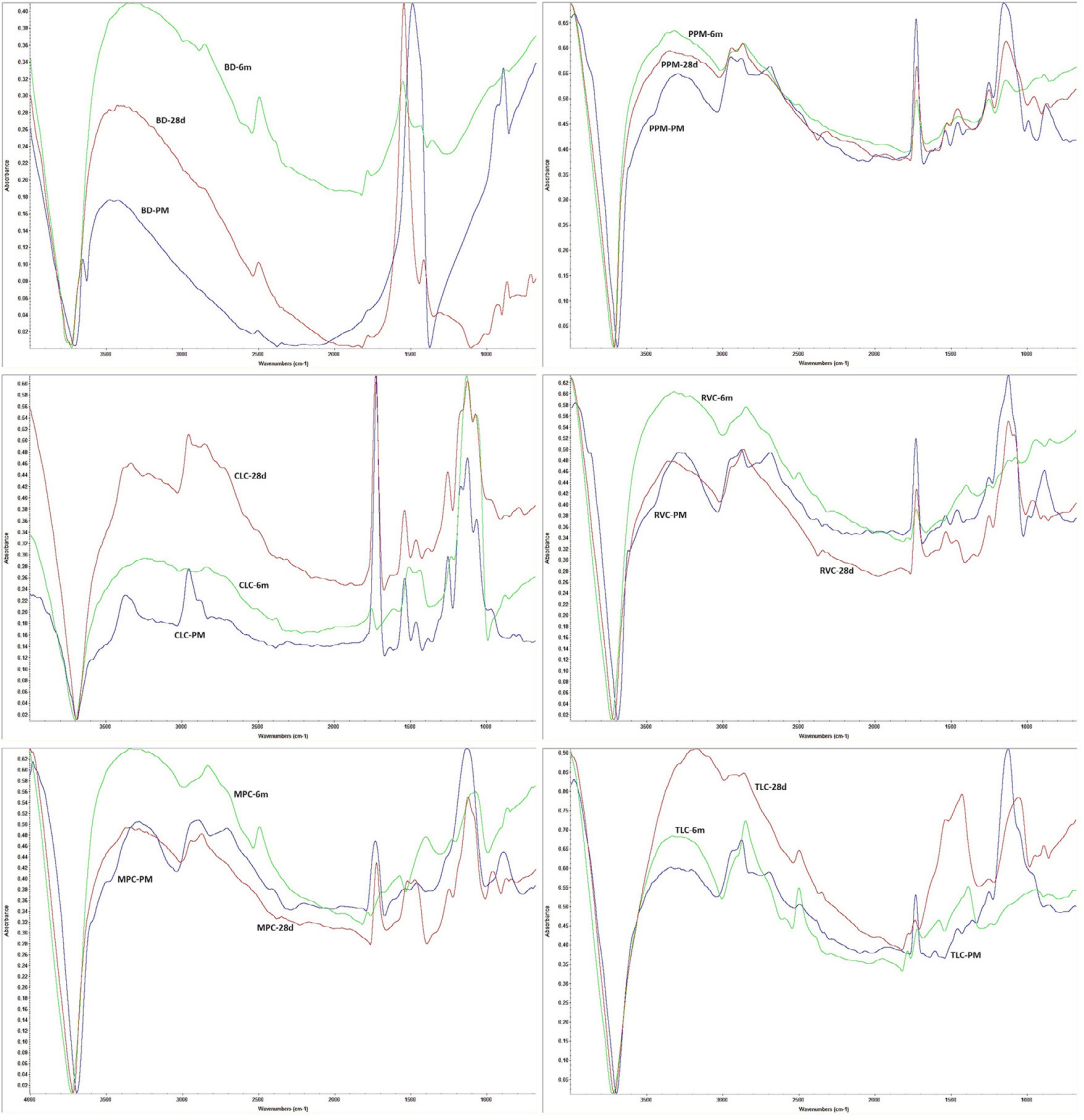
The sum spectra of the material samples’ Fourier transformation infrared spectroscopy analysis at baseline, 28 days, and 6 months (180 days). For all materials, n = 3 samples were examined per time point.

BD, according to the immersion tests described above, was the only material that exhibited an overall mass decrease over the 6 months of immersion in HBSS. This behaviour was also visible in FTIR microscopy. First, the video images recorded on the BD samples after 6 months of immersion in HBSS versus the video images recorded on the surface of the samples right after the preparation exhibited changes, meaning a rougher surface but also the appearance of some islands being visible with a brighter aspect. Comparing the FTIR spectra (Figure 9), major changes could be observed, consistent with the transformation of the components of the calcium silicates into calcium phosphates (apatite phase—especially the peak centred between 1000 and 1060 cm^–1^), but also carbonate could be clearly visualised (based on the peaks from ∼870 and 1420-1450 cm^–1^). According to the literature,^24^ these peaks are well correlated with the presence of the C_2_S-C_3_S specific to BD, along with a high amount of calcite. A similar behaviour could also be observed in TLC. When a similar behaviour was observed, most probably this meant a silicate to apatite transformation over the 6 months of immersion in HBSS, but a smaller amount of apatite. For CLC, MPC, PPM, and RVC, the immersion did not lead to a high amount of apatite phase as the intensity at ∼1020 to 1050 cm^–1^ was very low.

In conclusion, based on the compositional evolution of the samples over a 6-month immersion in HBSS, BD was able to generate the highest amount of apatite, as visible from EDS, XRD, and FTIR. Some reduction in hydroxyapatite formation capability was observed for LCPCMs, while in most cases the P peak in EDS was difficult to identify.

### Summary of results

The pH values of BD were higher than the pH values of all LCPCMs at all time points examined. RVC and MPC exhibited a similar evolution of the pH values as BD, but the difference was statistically significant at all time points examined (*P* < .05). The same applied to conductivity and solubility measurements. BD had a statistically significant higher conductivity than all LCPCMs at all examined time points (*P* < .05). BD was the only material examined exhibiting an overall loss of mass, while all LCPCMs gained mass, a statistically significant difference (*P* < .05). In the EDS, calcium was found on the surface of all samples, with BD having the highest concentration of calcium on the surface, and the difference between BD and the LCPCMs was statistically significant (*P* < .05). In the SEM, clear blossom-like structures were found extensively on the surface of BD, while the LCPCMs exhibited overall less surface activity, with crystalline structures forming in a reduced amount on their surface. Apatitic phases were recorded in the XRD for BD, CLC, MPC, PPM, and RVC and in the FTIR for BD and TLC after storage in HBSS for up to 180 days.

## Discussion

In the present study, the surface reactivity of 5 different LCPCMs containing either calcium hydroxide powder (CLC) or calcium silicate powder (TLC, RVC, MPC, and PPM) when stored in simulated body fluid (HBSS) has been investigated in comparison to BD. Among the LCPCMs, RVC, MPC, and PPM have not been investigated before. Therefore, this is the first time they have been included in a study. No other data are available on these materials’ behaviour in simulated body fluids to date.

To ensure the reliability of the results, a large number of samples were prepared. The samples were then kept under standardised conditions and constant movement in the HBSS. The in vitro activity of the samples was evaluated based on the evolution of the pH and conductivity of the HBSS solution over a long period of time, as well as based on the mass change and water absorption of the samples over the same time period. The morpho-structural and chemical composition of the surfaces was also investigated. In contrast to many studies available in the relevant literature, which usually only cover a period of 14 to 28 days, in this study, long-term results over a period of 6 months were generated. This is important because materials for VPT remain in the tooth for many years. The results of the present study are conclusive and consistent; none of the investigations contradicted the others. A limitation of the present study is the lack of biocompatibility investigations or antimicrobial investigations, as well as the lack of investigation of the elution of monomers. These must be further investigated in future studies.

Amongst the 6 materials investigated in this study, BD exhibited the highest pH value of the storage solution at all the investigated time points. The highest standard deviation at 1 day (±0.75), compared with that at 6 months, is evidence that BD is highly active immediately after immersion, and minor variability in processing leads to important changes. In the long term, though, the standard deviation is reduced consistently (to only ±0.13), which means that in the long term, the samples’ behaviour is very stable. For the LCPCM samples, the standard deviation is not dependent on the immersion time. Practically, the standard deviation is similar for different LCPCM materials at the same time, indicating that the release and deposition processes occur in a more controlled manner. The difference between the pH value of BD and all 5 investigated LCPCMs was statistically significant. The result of the present study is consistent with the relevant literature, where similar results have been obtained. LCPCMs induced a lower pH value of the storage fluid, regardless of whether they were stored in simulated body fluids such as HBSS or simulated body fluid (SBF)^17^^,^^18^ or stored in distilled water.^14^, ^15^, ^16^^,^^25^ It must be emphasised at this point that studies in which simulated body fluids were used as a storage medium are, from our perspective, more accurate, as there is a lower concentration gradient between the fluid and the material sample than when distilled water is used. Distilled water could draw more ions from the materials than would otherwise be the case due to the high concentration gradient. Furthermore, it is known from the relevant literature that HCSCs can form hydroxyapatite-like crystals on their surface when in contact with simulated body fluids.^26^^,^^27^ For this reason, simulated body fluid (HBSS) was used as the storage medium in the present study.

In vitro, by immersing LCPCMs in HBSS, these samples not only induce a lower pH value of the storage solution, meaning that they release less HO^–^ ions into the storage solution, but also release less calcium ions when stored in distilled water^23^ or simulated body fluids.^17^^,^^21^^,^^22^^,^^25^ In line with these results from the literature, our results confirmed this. This study found that the storage liquid of BD exhibited a significantly higher conductivity than that of the LCPCMs at any time point considered. The conductivity is an indicator of the amount of ions in the liquid. The more ions in the liquid, the higher the conductivity. A higher conductivity of BD therefore means that significantly more ions must be released from BD than from the LCPCMs. This is also reflected in the increased pH of BD’s storage solution. However, it is not only hydroxyl ions that are responsible for the increasing conductivity of the storage liquid of BD, as the curve for the pH value does not rise as quickly and as highly as the curve for the conductivity, meaning that calcium and silicate ions must also be released from BD. If this were not the case, no solubility would be detected for BD. In fact, the only samples with an overall negative mass change (where solubilisation was more important than water sorption via chemical and physical processes) were the BD samples. This also corresponds to the results of the EDS and XRD analysis in this study, where more calcium ions were detected on the surface of BD.

The present study found a higher solubility for BD than for LCPCMs. According to ISO 6876:2012,^28^ solubility of set materials should not exceed 3% mass fraction after 24 hours. BD reached a mass loss of 1.26% mass fraction after 6 months and 0.24% after 24 hours. Thus, the values determined correspond to the value set forth in ISO 6872:2012, and BD can be considered stable over a long period of time, a result also generated in other studies.^27^ The application of ISO 6876:2012 to rate the solubility of HCSCs has become established in the past and is the most common ISO standard used to evaluate HCSC in studies.^27^^,^^29^ This is because there is no separate ISO standard for HCSCs, but these materials, similar to root canal sealers, can come into contact with periapical tissue when used as root end sealing materials in an apical plug. It has therefore become established practice to also evaluate HCSCs using the ISO 6876:2012 standard. In contrast, for the LCPCMs, an overall positive mass change was recorded, meaning that the composites have water affinity. The highest recorded value was for MPC, with a mass gain of 5.18% mass fraction after 6 months, and the lowest recorded value was for PPM, with a mass gain of 1.13% mass fraction after 6 months. The difference between BD and all LCPCMs was statistically significant, consistent with the relevant literature. Other studies also report a lesser solubility of LCPCMs compared to HCSCs,^15^^,^^30^ but there are studies that report a higher solubility of LCPCMs compared to HCSCs.^31^ Strictly speaking, though, the present method quantifies the elution of water-soluble components rather than true solubility, which is defined as the thermodynamic equilibrium between a solid and its saturated solution.^32^ In addition, changes in specimen mass may reflect structural disintegration unrelated to dissolution, such as particle detachment from the cement matrix during storage,^32^^,^^33^ while water sorption may partially compensate for dissolution-related mass loss.^33^^,^^34^ Still, weight loss comes closest to solubility in the present experimental setting. Weight loss was assessed by measuring the reduction in specimen mass after liquid storage, as previously described.^33^^,^^35^ Although the International Standard recommends determining material loss via the residue method, meaning a mass increase of the storage dish,^28^ direct specimen weighing was performed to minimise underestimation of material released into solution. Each specimen was assigned to a single immersion period to avoid possible artefactual mass changes associated with repeated drying and reimmersion.

The surface analyses of the different materials were conclusive with the abovementioned findings. In the SEM examination, hydroxyapatite-like precipitates in the form of a blossom could be observed on the surface of the BD. These appeared after only 7 days and multiplied and grew into a multilayered carpet of blossom-like structures over the course of the investigation. The formation of such blossom-like structures on the surface of HSCS when in contact with simulated body fluid has been previously described.^36^ On the surface of the LCPCMs, however, little to no activity was seen in the form of blossom-like precipitates. Crystalline, jagged, salt-like precipitates and platelets overlying the surface were formed, which most closely corresponded to the calcite found in the XRD. Subsequently, isolated blossom-like structures formed on top of the crystalline platelets in the MPC but not in the form and quantity observed in BD. These findings correspond to other SEM investigation results in the relevant literature, which indicate that calcium silicate cements have a typical cement matrix with fully hydrated cement particles, while the LCPCMs consist of a composite resin matrix and unhydrated cement particles.^17^^,^^22^^,^^25^ The conducted EDS examination confirmed these findings. Significantly more calcium was detected on the surface of BD compared to LCPCMs. The sinking or varying values of calcium on the surface of BD over time in this study can be explained by the fact that over the course of the immersion in HBSS, calcium elutes from BD; thus, the value of calcium on the surface decreases. Macroscopically, a white salt precipitate was found in the sample tubes after 28 days and 6 months. These results are also consistent with another study, which reported decreasing values of calcium ion release from BD over a period of 28 days.^25^ The authors of that study report that hydroxyapatite forms on the surface of BD and LCPCMs,^25^ a finding that can be partially confirmed in the present study. Another EDS examination of the LCPCMs compared to the HCSCs showed that all materials contained calcium, silicon, aluminium, oxygen, and carbon, but the HCSCs contained significantly more calcium on their surface than the LCPCMs.^23^

In the XRD analyses, tricalcium silicate was detected in both LCPCMs and BD,^14^^,^^21^^,^^22^ as in the present study. No calcium hydroxide was detected in the LCPCMs, but it was detected in BD.^14^^,^^21^^,^^22^ These findings are consistent with the present study, in which calcite, probably from carbonated calcium hydroxide, and hydroxyapatite were found extensively on the surface of BD and, in a lesser amount, on the surface of LCPCMs. The apatite phase was obtained for BD, CLC, MPC, PPM, and RVC but not for TLC. This means that a certain surface activity in the form of apatite formation can be observed on the surface of the BD as well as on the surface of the LCPCM. This is consistent with the changes in pH value and conductivity described above and the observations made using the SEM and EDS. Calcite and tricalcium silicate, among others, were found on the surface of BD as well as the LCPCMs. In the XRD of BD, other studies also found silicon, calcium, oxygen, carbon, tricalcium silicate, calcium carbonate, and portlandite,^21^ and while BD had a surface covered by calcium and silicon particles, the surface of LCPCM was bare and uncovered. This finding corresponds to the findings of the present study. Others found tricalcium silicate only in the HCSCs, while the LCPCMs contained only calcium oxide.^23^

The conducted FTIR analysis revealed peaks for apatitic phases, carbonates, and di- and tricalcium silicates for BD. For the LCPCMs, more specifically for TLC, a lesser amount of apatite was evident in the FTIR examination. For CLC, MPC, RVC, and PPM, no apatitic phases were detected. The LCPCMs exhibited typical bands for resin composites, pointing to their polymeric content. These bands were visible even after long-term immersion in HBSS, with the intensity changes being more a consequence of surface activity than of dissolution/depolymerisation, as, based on the FTIR analysis, we were not able to assess the presence of these compounds in the solution. Still, because of the low sensitivity of this technique, further chromatographic assessments must be considered to evaluate such a release. These findings are consistent with other studies.^25^ Although some authors report the detection of calcium hydroxide in LCPCMs after immersion in phosphate buffered saline in the FTIR examination,^25^ they also report that in Raman spectroscopy, they detected more intense peaks for calcium silicate on the surface of freshly mixed BD than on the surface of LCPCMs and that only BD exhibited calcite on its surface.^25^ While in the present study, apatite and hydroxide in a carbonated form were mainly detected on the surface of BD, other authors reported that after 7 days of immersion in SBF,^31^ as well as after 3, 14, and 28 days of immersion in SBF, they recorded a hydroxyapatite-specific peak in the FTIR examination of LCPCMs,^37^ as well as bands for apatite, calcite, and phosphate from apatite.^37^ Still, more detailed examinations of the morphologic and exact chemical changes and the composition of the precipitates on the surface of LCPCMs need to be conducted to better understand this material class.

The moderate changes on the sample surface of the LCPCMs in the SEM, EDS, XRD, and FTIR analysis and the inferior results regarding the pH change and conductivity of the HBSS, as well as the positive mass change of the LCPCMs, which altogether may explain their lower activity, are an indicator that their use is questionable. If one now additionally bears in mind that the relevant literature with regard to the biocompatibility of LCPCMs is mostly not in favour of composite-resin materials, as they release monomers,^23^^,^^38^ exhibit clear cytotoxic properties in vitro,^39^^,^^40^ and have little or no mineralisation-inducing effects in vitro,^40^^,^^41^ it must be critically scrutinised whether these materials are suitable for VPT. This hypothesis is supported by meta-analyses of comparative clinical studies, which found that HCSCs such as Biodentine or MTA perform significantly better than LCPCMs such as TheraCal LC or LC Calcihyd^42^ or calcium hydroxide^43^ when used for direct pulp capping.

## Conclusion

The results of this study indicate that the investigated LCPCMs are inferior to BD in terms of the material science parameters examined here. Although certain LCPCMs show a pH evolution comparable to BD, the pH values of BD differ significantly from those of all LCPCMs at every time point examined. BD demonstrates significantly higher conductivity throughout the entire observation period, and BD is the only material tested with an overall negative mass change, which also represents a statistically significant difference at all time points examined. Surface analyses revealed less pronounced morphologic alterations on the surface of all LCPCMs, with apatitic phases being detected by XRD and FTIR only to a limited extent, indicating that LCPCMs have a lesser ability to develop hydroxyapatite-like structures on their surface than BD when in contact with simulated body fluids.

## Author contributions

Laurentia Schuster: conceptualisation, methodology, data curation, formal analysis, investigation, methodology, writing – original draft. Till Dammaschke: conceptualisation, writing – review & editing, supervision. Roxana Trusca: investigation and data curation. Ludmila Motelica: investigation. Benjamin Ehmke: supervision. Denisa Ficai: investigation, writing – review & editing. Anton Ficai: conceptualisation, methodology, project administration, supervision, validation, writing – review & editing

## Funding

This research did not receive any specific grant from funding agencies in the public, commercial, or not-for-profit sectors.

## Conflict of interest

The authors declare the following competing financial interests or personal relationships that could have appeared to influence the work reported in this paper: Till Dammaschke claims to have received fees from Septodont for lectures. All other authors declare no conflict of interest.
